# Nanocomposite Film Development Based on Chitosan/Polyvinyl Alcohol Using ZnO@Montmorillonite and ZnO@Halloysite Hybrid Nanostructures for Active Food Packaging Applications

**DOI:** 10.3390/nano12111843

**Published:** 2022-05-27

**Authors:** Aris E. Giannakas, Constantinos E. Salmas, Dimitrios Moschovas, Maria Baikousi, Eleni Kollia, Vasiliki Tsigkou, Anastasios Karakassides, Areti Leontiou, George Kehayias, Apostolos Avgeropoulos, Charalampos Proestos

**Affiliations:** 1 Department of Food Science and Technology, University of Patras, 30100 Agrinio, Greece; gkechagi@upatras.gr; 2 Department of Material Science and Engineering, University of Ioannina, 45110 Ioannina, Greece; dmoschov@uoi.gr (D.M.); mbaikou@uoi.gr (M.B.); tasos.karakassides@gmail.com (A.K.); aavger@uoi.gr (A.A.); 3 Laboratory of Food Chemistry, Department of Chemistry, National and Kapodistrian University of Athens Zografou, 15771 Athens, Greece; elenikollia@chem.uoa.gr (E.K.); vtsigkou@chem.uoa.gr (V.T.); 4 Laboratory of Food Technology, Department of Business Administration of Agricultural and Food Enterprises, University of Patras, 30100 Agrinio, Greece; aleontiu@upatras.gr

**Keywords:** active packaging, nanostructure, nanocomposite, ZnO, montmorillonite, halloysite, chitosan, polyvinyl alcohol, antimicrobial activity

## Abstract

The global turn from the linear to the circular economy imposes changes in common activities such as food packaging. The use of biodegradable materials such as polyvinyl alcohol, natural raw materials such as clays, and food byproducts such as chitosan to develop novel food packaging films attracts the interest of industrial and institutional research centers. In this study, novel hybrid nanostructures were synthesized via the growth of zinc oxide nanorods on the surface of two nanoclays. The obtained nanostructures were incorporated with chitosan/polyvinyl alcohol composite either as nanoreinforcement or as an active agent to develop packaging films. The developed films were characterized via XRD, FTIR, mechanical, water-vapor diffusion, water sorption, and oxygen permeability measurements. Antimicrobial activity measurements were carried out against four different pathogen microorganisms. XRD indicated the formation of an intercalated nanocomposite structure for both types of nanoclays. Furthermore, improved tensile, water/oxygen barrier, and antimicrobial properties were recorded for all films compared to the pure chitosan/polyvinyl alcohol film. Overall, the results indicated that the use of the bio-based developed films led to an extension of food shelf life and could be used as novel active food packaging materials. Among them, the most promising film was the 6% wt. ZnO@halloysite.

## 1. Introduction

Nowadays, following the spirit of sustainability, cyclic economy, and green chemistry, the food industry has turned to the development of novel added-value functional packaging materials using food by-products. As a result, there is an increasing interest in replacing the use of fuel-based polymers with the use of novel developed bio-based polymers and thus moving to the basis of more sustainable materials. Chitin [1,2] is the second most abundant biopolymer after cellulose. It consists of 2-acetamido-2-deoxy-β-d-glucose through a β (1→4) linkage. The exoskeletons of arthropods such as crustaceans and insects, the cell walls in fungi, the beaks of cephalopod, and the radulae of mollusks are some major natural sources of this substance. Chitosan (CS) is the *N*-deacetylated derivative of chitin [2,3,4]. The deacetylation process of chitin to chitosan (CS) can be obtained through a quite simple thermal treatment with NaOH [5,6]. Nowadays, special treatment of crab or shrimp shells and fungal mycelia is one of the famous manners of obtaining chitin. Thus, food industries such as shrimp canning are one of the major sources of raw material for chitin and CS production [2,6]. CS is a biodegradable, non-toxic polymer derived from renewable resources and exhibits a great potential to be applied as a packaging material [7].

To enhance the tensile and barrier properties of biopolymer-based packaging films, the addition of various nanofillers is suggested [8,9]. Layered silicate nanoclays such as sodium montmorillonite (Mt) and halloysite (HNT) nanotubes are among the most promising materials among various nanofillers [10,11,12]. The addition of small amounts of nanofillers such as nanoclays into biopolymers gives rise to a new class of bio-nanocomposite-based packaging films [13,14]. In the last decade, much effort has been devoted to developing nanocomposite films based on CS/Mt [15,16,17,18,19,20] and CS/HNT [11,12,21,22,23,24,25] for food packaging and drug delivery applications. Recently, polyvinyl alcohol (PVOH) was added to a CS/Mt matrix, acting as an effective plasticizer and enhancing the barrier properties [26,27] as well as the antimicrobial properties [28].

Another attractive nanofiller due to its low cost, antibacterial properties, and UV-adsorption ability is ZnO. One of the materials registered by the US Food and Drug Administration (FDA) (FDA, 2011) in the Generally Recognized As Safe (GRAS) list is ZnO. The following mechanism steps were proposed to explain the antimicrobial activity of ZnO: (a) the release of Zn^2+^ antimicrobial ions via the dissolution of ZnO nanoparticles, (b) the penetration of cell membrane by nano-sized particles and the damage to the cell membrane, and (c) the destruction of the bacterial cells by the highly active oxygen species formed on the surface of the ZnO nanoparticles via irradiation [29,30]. In this study, the Zn^2+^ ion release could be beneficial for antimicrobial activity when ZnO nanoparticles encapsulated in acidic biopolymers carriers such as CS are diluted in acetic acid. In this case, the obtained CS/ZnO edible nanocomposite could be applied as both antimicrobial and nutritional Zn^2+^ ion agents [31,32]. Potential synergies between zinc oxide (ZnO) and chitosan could be exploited to develop active films/coatings with enhanced antimicrobial performance and biocapability [29,33,34,35,36]. The use of ZnO/clay nanostructures for photocatalytic applications is well known in the literature [37]. Recently, a growing interest was raised [38,39] for the development and application of such ZnO/clay nanostructures and their use as active food packaging materials. According to this simple and innovative idea, the ZnO is expected to act either as an antimicrobial or reinforcement agent, and nanoclays are expected to act as both a reinforcement and a barrier agent [39]. Ding et al. [38] prepared palygorskite@ZnO nanorods and loaded different contents into chitosan-based films. Their study shows that addition of palygorskite@ZnO nanorods enhanced the tensile strength and the antimicrobial properties of obtained films.

In a recent study [39], novel ZnO@Mt hybrid nanostructures, similar to one of the two reported in this study, were developed to achieve a novel nanofiller with enhanced tensile, barrier, and antimicrobial properties. Detailed structural characterization was carried out in this study, including SEM images. It is obvious from such images that the ZnO nanorods were grown on the surface of the clay. These ZnO@Mt nanostructures were prepared in various ZnO/Mt nominal wt% ratios and were added as nanofillers in polyvinyl alcohol biodegradable films. The overall study concluded that four times higher ZnO weight than the Mt weight is the optimum wt% ratio and exhibits optimum properties. By following this effort to develop biodegradable “green” polymers and to achieve higher shelf life for foods, in this work, we developed two different hybrid nanostructures considering the conclusion of this previous study. Considering the results of [39], in the case of the ZnO@Mt and the ZnO@HNT nanohybrids, the ZnO nanorods grow on the surface of the Mt and the HNT nanotubes, respectively. In both cases, the nominal wt% ratio of ZnO to nanoclays was kept constant and equal to 4. Each one of these nanohybrids was added into chitosan/polyvinyl alcohol (CS/PVOH) matrices in 3 and 6 wt% content to develop novel CS/PVOH/xZnO@Mt and CS/PVOH/xZnO@HNT nanocomposite edible active packaging films. Both the ZnO@Mt and ZnO@HNT nanohybrids, as well as the obtained CS/PVOH/xZnO@Mt and CS/PVOH/xZnO@HNT films, were characterized via XRD analysis and FTIR spectrometry. Mechanical properties, water/oxygen barrier properties, and antimicrobial activity against four different food pathogens of all obtained films were evaluated. The specific targets of this study were (1) the comparison/evaluation of the ZnO@NaMt or ZnO@HNT nanostructures’ performance as antimicrobial and nanoreinforcement components in the development of CS/PVOH edible films and (2) the comparison between these two materials.

## 2. Materials and Methods

### 2.1. Materials

Acros-Organics company (Zeel West Zone 2, Janssen Pharmaceuticalan 3a, B2440, Geel, Belgium) was the provider of chitosan with a molecular weight of 100,000–300,000. SIGMA-ALDRICH, Co., 3050 Spruce Street, St. Louis, MO 63103 USA 314-771-5765 was the supplier of poly(vinyl alcohol) (PVOH) with low molecular weight (13,000−23,000) and hydrolysis degree of 87−89%. The same company was also the provider of zinc acetate dihydrate (Zn(CH_3_COO)_2_·2H_2_O), 25% ammonia solution, and montmorillonite (Mt) (code name Nanomer PGV) with a mass density of 2.6 g/cm^3^ and CEC value of 145 meq/100 g. The last chemical was purchased from Nanocor Inc., 2870 Forbs Avenue Hoffman Estates, IL 60192, IL, United States. Sigma-Aldrich was the supplier of the montmorillonite (product 685445, Sigma-Aldrich, St. Louis, MO, USA) with a chemical composition of 1.53% halloysite nanotubes (Al_2_Si_2_O_5_(OH)_4_·2H_2_O, 99.5% clay), 1.68% CaO, 3.35% Fe_2_O_3_, 62.9% SiO_2_, 19.6% Al_2_O_3_, and 3.05% MgO.

#### 2.1.1. Preparation of ZnO@Mt and ZnO@HNT Hybrid Nanostructures

In this work, ZnO nanorods were developed after 1 h hydrothermal treatment of Zn acetate solution. This result is well supported by literature reports [39,40]. In all cases, the nominal amount of 2 g ZnO nanorods was obtained using 4.525 g of Zn(CH_3_COO)_2_·2H_2_O (24.7 mmol). The amount of the Mt and HNT which was used to obtain the final ZnO@Mt and ZnO@HNT nanohybrids was 0.5 g, and the ZnO to nanoclay mass fraction was equal to 4. Briefly, 50 mL aqueous solution was prepared by dissolving 4.525 g of Zn(CH_3_COO)_2_·2H_2_O (24.7 mmol) in deionized water and stirred for 5 min. A white precipitate slurry was initially observed. Then a 25% w/w aqueous solution of NH_3_ was added drop by drop to the above Zn(CH_3_COO)_2_·2H_2_O aqueous white slurry under constant stirring. The pH was adjusted to ~11. The addition of the NH_3_ (approx. 11 mL) caused the dissolution of the white precipitate. Finally, appropriate amounts of Mt and HNT were spread into the transparent solution and further stirred for 2 h. ZnO@Mt and ZnO@HNT precipitates were obtained after 1 h aging, reflux, and several times washing of slurries with deionized water to remove ammonia excess. The final product was dried at 60 °C for 24 h.

#### 2.1.2. Preparation of CS/PVOH/xZnO@Mt and CS/PVOH/xZnO@HNT Active Films

**Figure 1:**
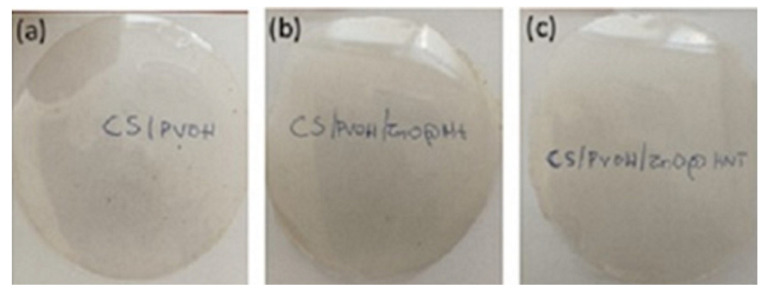
Representative photos of (**a**) pure CS/PVOH film and (**b**) CS/PVOH/6ZnO@Mt and (**c**) CS/PVOH/6ZnO@HNT nanocomposite films.

Reflux and heated press are two of the multiple steps followed to prepare all films. The overall procedure is well described in our previous publications [16,26,41,42]. Firstly, 20 g of CS was dispersed in 1000 mL aqueous solution with 1% *v*/*v* Hac and was stirred vigorously for 24 h at 70 °C. The obtained 2% *w*/*v* CS solution (pH 4.4) was stored at room temperature until it cooled down. Appropriate amounts of 2% *w*/*v* CS solution and hot PVOH aqueous solution were mixed to obtain 20% *w*/*w* PVOH content in final blends. The mixtures were stirred and refluxed under stirring for 2 h. The obtained CS/PVOH solution was cast onto 12 cm diameter plastic dishes and dried for ∼5 days at ambient conditions (∼22 °C). The obtained pure CS/PVOH film was peeled off and pressed under 3 MPa constant pressure at 120 °C for 2 min, using a hydraulic heated press. Appropriate amounts of ZnO@Mt and ZnO@HNT nanohybrids were added to the CS/PVOH solution to prepare the CS/PVOH/clay nanocomposite films with 3 wt% and 6 wt% nanohybrid content. The solutions with the dispersed ZnO@clay materials were refluxed under stirring for 4 h. The obtained liquids were treated in the same way as described above for the CS/PVOH solution. The code names of the materials and the exact quantities used for the preparation of the films are presented in Table 1. Representative photos of pure CS/PVOH, CS/PVOH/6ZnO@Mt, and CS/PVOH/6ZnO@HNT nanocomposite films are shown in Figure 1. It is obvious from these photos that the addition of ZnO@Mt and ZnO@HNT nanostructures in the CS/PVOH matrix does not significantly affect the transparency of obtained films. This shows the high dispersity of both ZnO@Mt and ZnO@HNT nanostructures in the CS/PVOH matrix, which is desirable and indicative of the existence of a nanocomposite structure.

### 2.2. XRD Analysis

For the analysis of the structural morphology of both ZnO@Mt and ZnO@HNT powder nanostructures, as well as that of the developed CS/PVOH, CS/PVOH/xZnO@Mt, and CS/PVOH/xZnO@HNT films, a Brüker D8 Advance X-ray diffractometer (Brüker, Analytical Instruments, S.A., Athens, Greece) was used. The diffractometer was equipped with a LINXEYEXE High-Resolution Energy-Dispersive detector.

### 2.3. FTIR Spectrometry

The chemical structure of both ZnO@Mt and ZnO@HNT hybrids was confirmed by IR spectra measurements. The measurements were carried out with an FT/IR-6000 JASCO Fourier transform spectrometer (JASCO, Interlab, S.A., Athens, Greece). For all measurements, the frequency range was 4000 to 400 cm^−1^. The obtained infrared (FTIR) spectra of each measurement were the average of 32 scans at 2 cm^−1^ resolution.

### 2.4. SEM Images

The surface morphology of the obtained ZnO@Mt and ZnO@HNT nanohybrids, as well as that of the representative CS/PVOH/6ZnO@Mt and CS/PVOH/6ZnO@HNT nanocomposite films, was investigated using a JEOL JSM-6510 LV SEM Microscope (Ltd., Tokyo, Japan) equipped with an X-Act EDS-detector from Oxford Instruments, Abingdon, Oxfordshire, UK (an acceleration voltage of 20 kV was applied). EDX measurements were also carried out.

### 2.5. Tensile Properties

Tensile measurements were carried out for all obtained CS, CS/PVOH, CS/PVOH/xZnO@Mt, and CS/PVOH/xZnO@HNT films by using a Simantzü AX-G 5kNt instrument (Simandzu. Asteriadis, S.A., Athens, Greece). Such measurements were carried out according to the ASTM D638 method. Three to five “dog-bone” samples (with gauge dimensions of 10 × 3 × 0.22 mm) of each film were tensioned at a cross-head speed of 2 mm/min. Force (N) and deformation (mm) values were recorded during the test, and the stress–strain curves were plotted. Modulus of elasticity, tensile strength, and strain at break values were calculated by using these stress–strain curves and the appropriate software for the instrument.

### 2.6. Water Sorption

Small pieces (20 × 20 mm) of the selected films were placed under vacuum in a desiccator for 24 h. Their dry mass was determined by weighing. The weighed films were stored in closed beakers at T = 25 °C with 50 mL of deionized water. Periodical weighing of samples enables the calculation of the total water sorption value at the saturation with water point. The equation for such calculations is as follows:

(1) $$W.G.\left(\%\right)=\frac{m_{\mathrm{wet}}-m_{\mathrm{Dry}}}{m_{\mathrm{Dry}}}\times 100$$

where m_Wet_ and m_Dry_ are the weights of the wet and dry films, respectively, and W.G. is the water gain.

### 2.7. Water Vapor Diffusivity

Water vapor transmission rate (WVTR) through active films was measured at 38 °C and 50% RH. Such measurements were carried out for all CS/PVOH/xZnO@Mt and CS/PVOH/xZnO@HNT materials according to the ASTM E96/E 96M-05 method. The procedure and the handmade apparatus for the WVTR measurements are reported in previous publications [16,17,43,44,45]. On the top of a one-open-end cylinder with approx. 10 cm length, film discs with approx. 2.5 cm diameter and approx. 0.15 mm thickness were placed and sealed by a rubber O-ring. The cylinder, which contained dried silica gel, was placed in a glass desiccator with 200 mL saturated magnesium nitrate solution. This solution produces 50% atmospheric relative humidity (RH) at 38 °C. Periodically weighing containers for 24 h enables the calculation of the mean WVTR according to the following equation:

(2) $$\mathrm{WVTR}=\frac{G}{t}\times \frac{1}{A}$$

where G is the weight gain of the cylinder in grams, t is the weighing time in hours, G/t is the slope of the fitted straight line on the experimental ΔG vs. t points, and A is the permeated cross-sectional area of the film. Furthermore, tested films were weighed before and after the WVTR measurements to exclude the humidity amount that could be absorbed by the film.

Water vapor diffusion coefficient through films (D_WV_) was calculated following Fick’s law principles and according to Equation (3), the derivation of which was described in detail in our previous publication [28].

(3) $$D_{\mathrm{WV}}=\mathrm{WVTR}\;\times \frac{\Delta x}{\Delta C}$$

where D_WV_ (cm^2^ · s^−1^) is the diffusion coefficient, WVTR (g · cm^−2^ · s^−1^) is the water vapor transmission rate, Δx (cm) is the mean film thickness, and ΔC (g · cm^−3^) is the humidity concentration gradient in the two opposite sides of the film, which in our case is 22.86747 g · cm^−3^ according to the calculations reported in our previous publication [28].

### 2.8. Oxygen Permeability

An oxygen permeation analyzer (O.P.A., 8001, Systech Illinois Instruments Co., Johnsburg, IL, USA) was used to measure the oxygen transmission rate (OTR). The selected experimental conditions for these measurements were 23 °C and 0% RH according to the ASTM D 3985 method. OTR values were measured in cc O_2_/m^2^/day, and the oxygen permeability coefficient values (Pe_O2_) were calculated according to Equation (4). The OTR value of each kind of film was the mean value of measurements on three pieces. The origination of Equation (4) is described in detail in our previous publication [28].

(4) $${\mathrm{Pe}}_{O2}=\mathrm{OTR}\;\times \;\Delta x$$

where Pe_O2_ (cm^2^ · s^−1^) is the oxygen permeability coefficient through films, OTR (cm^3^ · m^−2^ · s^−1^) is the measured oxygen transmission rate, and Δx (cm) is the mean film thickness.

### 2.9. UV–Vis Transmittance Analysis of Films

UV–vis absorbance measurements were carried out for the pure CS/PVOH, the CS/PVOH/6ZnO@Mt, and the CS/PVOH/6ZnO@HNT active nanocomposite films. Measurements were carried out with a Shimadzu 1900 spectrophotometer in the range of 200 to 800 nm.

### 2.10. Antimicrobial Activity Tests

The agar diffusion method was followed to estimate the antimicrobial activity of the films against four foodborne pathogenic bacteria, i.e., Gram-negative bacteria *Escherichia coli* (ATCC 25922), *Salmonella enterica subsp*. *Enteric* (DSMZ 17420), Gram-positive bacteria *Staphylococcus aureus* (DSMZ 12463), and *Listeria monocytogenes* (DSMZ 27575). The Institute of Technology of Agricultural Products, ELGO-DEMETER, Lykovryssi, Greece, was the supplier of the tested microorganisms.

A final concentration in the range of 10^7^–10^8^ CFU mL^−1^ was achieved by culturing bacterial strains in Mueller–Hinton broth at 37 °C for 24 h incubation. Next, the bacteria were grown on Mueller–Hinton agar dishes. Rotation of the plate 60° every time ensured the homogeneity of the growth.

Sample films of 6 mm diameter were sterilized using a UV lamp and placed on Mueller–Hinton inoculated dishes. Incubation was carried out at 37 °C for 24 h. The diameters of inhibitory zones and the contact area of the discs with agar surface were measured. The experiment was performed three times.

## 3. Results

### 3.1. XRD

**Figure 2:**
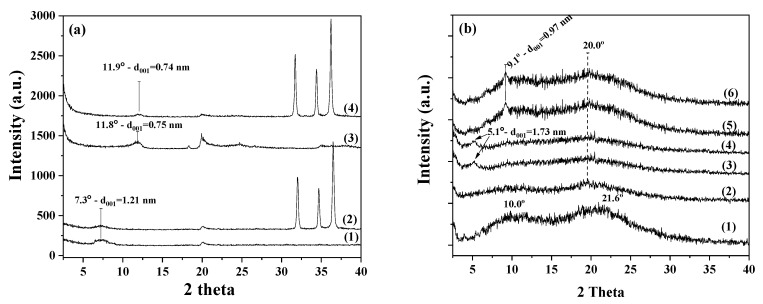
XRD plots of (**a**) pure Mt (1), ZnO@Mt nanohybrid (2), pure HNT (3), and ZnO@HNT nanohybrid (4) and (**b**) pure CS film (1), CS/PVOH film (2), CS/PVOH/3ZnO@Mt film (3), CS/PVOH/6ZnO@HNT film (4), CS/PVOH/3ZnO@Mt film (5), and CS/PVOH/6ZnO@HNT film (6).

Figure 2a shows the X-ray diffraction patterns of pure Mt, pure HNT, ZnO@Mt, and ZnO@HNT nanohybrids in a 2theta angle range from 2theta = 2° to 2theta = 40°. The XRD peaks at 2theta angles around 31.7°, 34.4°, and 36.2° correspond to the (100), (002), and (101) reflections of hexagonal:P63mc zinc oxide wurtzite crystal phase (COD-2015 library, Crystallography Open Database 139). The (002) reflections originated from the vertically oriented ZnO nanowires, while the (101) reflections originated from the tilted nanowires. The increase in the (002) peak indicates well-oriented ZnO nanowires [28,31,32]. These peaks, which were depicted in all XRD plots of the ZnO@Mt nanohybrids, indicated the growth of ZnO nanorods on Mt [46,47]. Using the theory of Williamson and Hull [48] and the methodology described elsewhere [49], the crystal size of ZnO nanorods was calculated and found to be 64.9 nm and 37.5 nm for ZnO@Mt and ZnO@HNT nanohybrids, respectively. Crystal size is known to be crucial for the antimicrobial activity of such ZnO nanorods [50].

Additionally, in all XRD plots of ZnO@Mt and ZnO@HNT nanohybrids, the basal spacing (d_001_) of Mt and HNT was observed at the low angle region around 7.3° and 11.9°, respectively. Comparing the basal space of the pure Mt with the corresponding one of the ZnO@Mt and the basal space of the pure HNT with the corresponding one of the ZnO@HNT, no changes were observed. These facts indicate that no changes were taking place in the interlayer space of the Mt and the HNT after the ZnO growth. This result is in accordance with the results reported in our previous work [39], and it shows that, by following the same preparation procedure, the ZnO nanorods grow on the external surface of either the Mt or HNT nanoclays.

Figure 2b shows the XRD patterns of the pure CS film and CS/PVOH blend film. It also shows representative XRD plots of the CS/PVOH/nanoclay nanocomposite films. The pure CS sample exhibits two wide reflections, at around 10° and around 21.6°. This pattern indicates the existence of small and imperfect crystals [51,52]. In the case of the CS/PVOH blend film, the peak of neat CS at a 2theta angle around 10° almost disappears. This peak shift is indicative of chain interactions between CS and PVOH and suggests that the PVOH molecules expand the free space in the CS chain [26,53].

The addition of Mt or HNT clay nanofiller into the CS/PVOH blend leads to a shift of 001 reflection from 7.3° to 5.2° for ZnO@Mt and from 11.9° to 9.3° for the ZnO@HNT sample. These shifts in lower angles indicate the formation of intercalated nanocomposite structures for both CS/PVOH/6ZnO@Mt and CS/PVOH/6ZnO@HNT nanocomposite film samples. Furthermore, XRD patterns of both CS/PVOH/xZnO@Mt and CS/PVOH/xZnO@HNT nanocomposite films exhibit too low characteristic peaks at 2theta angles 31.7°, 34.4°, and 36.2°, which are representative of the (100), (002), and (101), reflections of the hexagonal:P63mc zinc oxide wurtzite crystal phase. This indicates the transformation of ZnO nanoparticles into Zn^+2^ ions inside the acidic CS matrix.

### 3.2. FTIR

**Figure 3:**
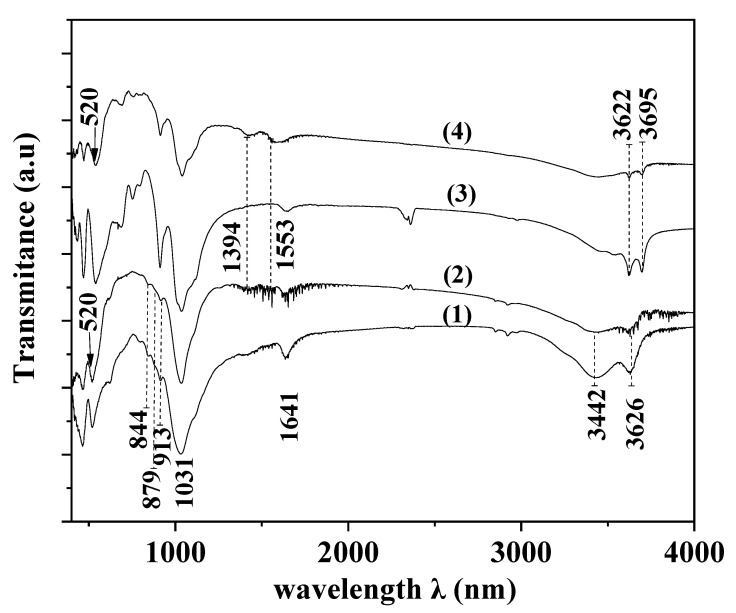
FTIR plots of pure Mt (1), ZnO@Mt nanohybrid (2), pure HNT (3), and ZnO@HNT nanohybrid (4).

Figure 3 shows the FTIR spectra of all ZnO@Mt and ZnO@HNT nanohybrids as well as the FTIR spectra of pure Mt and pure HNT. Mt nanoclay exhibits a characteristic absorption band at ~3626 cm^−1^ which indicates the stretching of the OH group bonded with the Al^3+^ cation [39,54]. It also exhibits a characteristic band at ~3442 cm^−1^ which is assigned to the H_2_O stretching vibrations. The band at ~1641 cm^−1^ is attributed to the H_2_O bending vibrations, while the bands at ~1113 cm^−1^ and 1031 cm^−1^ indicate the SiO stretching vibrations [55]. Furthermore, the peaks at 913, 879, and 844 cm^−1^ are bands of OH bending modes, while the band at ~913 cm^−1^ indicates the AlAl–OH bending mode. The bending mode of AlFe–OH is represented by the band at ~879 cm^−1^, while the bending mode of FeFe–OH is depicted by the band at ~844 cm^−1^ [56].

The band at 3695 cm^−1^ of the HNT FTIR spectrum indicates the stretching vibration of the inner surface OH groups. The stretching vibration of the inner groups is represented by the band at 3622 cm^−1^. Hydrogen bonds were formed between the oxygen sheets and the inner surface OH groups. Such OH groups are also connected to the Al-centered octahedral sheets. Two bands that are typical of inner surface OH groups of halloysite and should occur at approximately 3650 cm^−1^ and 3670 cm^−1^ cannot be observed [57].

ZnO@Mt and ZnO@HNT nanohybrids’ spectra exhibit an absorption band at around 520 cm^−1^. This band is typical and characteristic of the pure ZnO hexagonal wurtzite phase [58,59]. The absorption band at 3434 cm^−1^ indicates the O–H mode [60]. Furthermore, Mt and HNT peaks in both ZnO@Mt and ZnO@HNT nanohybrids’ FTIR spectra were reduced. This fact indicates the formation of ZnO nanorods on the external surface of Mt and HNT. At 1553 cm^−1^ and 1394 cm^−1^, two strong peaks existed, indicating the symmetric stretching of the carboxylate group (COO-) which originates probably from a small residue of zinc acetate that was used for the growth reaction [59].

### 3.3. SEM Images

**Figure 4:**
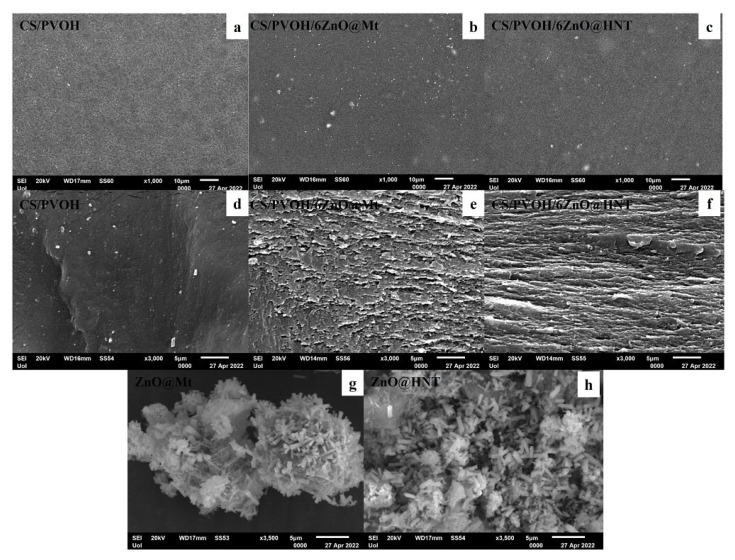
SEM images of surface and cross-section for the films: (**a**,**d**) CS/PVOH, (**b**,**e**) CS/PVOH/6ZnO@Mt, and (**c**,**f**) CS/PVOH/6ZnO@HNT. Surface images of nanohybrids (**g**) ZnO@Mt and (**h**) ZnO@HNT. SEM images.

**Figure 5:**
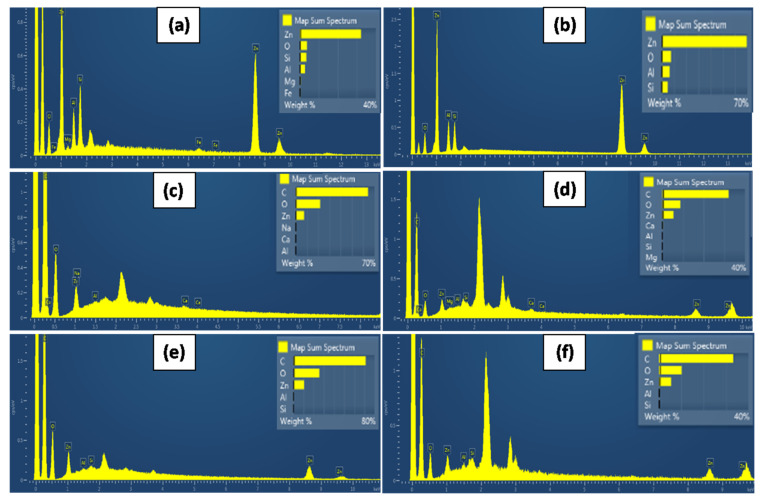
SEM-EDX images and elemental analysis of (**a**) ZnO@Mt and (**b**) ZnO@HNT nanohybrid powders, of CS/PVOH/ZnO@Mt (**c**) surface and (**d**) cross-section, and of CS/PVOH/ZnO@HNT (**e**) surface and (**f**) cross-section.

Figure 4 shows the surface (a, b, and c) and cross-section (d, e, and f) topology of the pure CS/PVOH film and the CS/PVOH/6ZnO@Mt and CS/PVOH/6ZnO@HNT active nanocomposite films after the incorporation of ZnO@Mt (g) and ZnO@HNT (h) nanohybrids in the content of 6%, respectively. The results of the SEM studies of the final nanocomposite films confirmed that the nanohybrids were homogeneously dispersed and indicated their significantly enhanced compatibility with the polymer matrix. The structure after the cross-section studies of the raw CS/PVOH surface matrices is smoother when compared to the respective nanocomposite films CS/PVOH/6ZnO@Mt and CS/PVOH/6ZnO@HNT which appear to be slightly rough with homogeneous morphology. From the (g) and (h) photos, it is obvious that the visible nanorod length falls in the range of 800–3000 nm, and it is safe to conclude that there are other smaller pieces. Thus, the ZnO crystallite size is well smaller, and the sizes calculated by the XRD measurements (i.e., 64.9 nm for the ZnO@Mt and 37.5 nm for the ZnO@HNT) are realistic. Moreover, it is safe to conclude that the ZnO nanorods are not visible in photos (b), (c), (e), and (f) because of the reduced sizes or the Zn^2+^ ions from the homogeneous dissolution in the CS/PVOH matrix. The existence of such components is evidenced by the EDX measurements which are presented in Figure 5.

Figure 5 depicts EDX images and elemental analysis for the ZnO@Mt and the ZnO@HNT nanostructures as well as for the CS/PVO/ZnO@Mt and CS/PVOH/ZnO/HNT films. It is obvious from the (a) and (b) photos that the growth of ZnO nanorods is over double in the case of the HNT clay (i.e., ~70% Zn) than in the case of the Mt clay (i.e., 32% Zn). It is also obvious from the (c) and (e) surface and (d) and (f) cross-section analysis that the Zn element exists inside the very well homogenized films, i.e., around 10% Zn estimated from the surface mapping and around 5% Zn estimated from the cross-section mapping. This strongly supports the results of the antimicrobial measurements which show the enhanced antimicrobial activity of the ZnO@HNT materials compared to the relevant ZnO@Mt materials.

**Figure 6:**
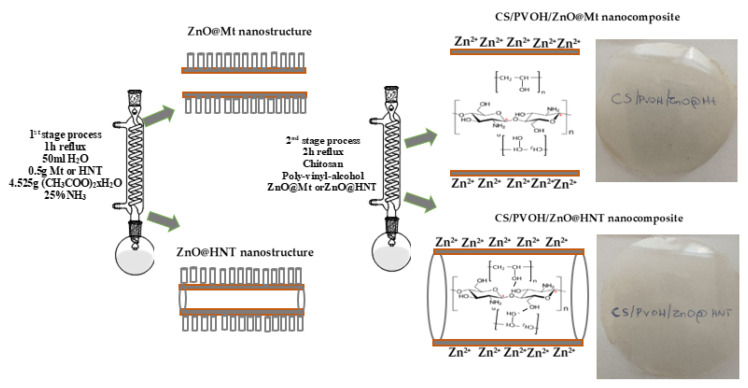
Illustration of the ZnO@Mt and ZnO@HNT nanohybrids and the formation process of the CS/PVOH/xZnO@Mt and CS/PVOH/xZnO@HNT intercalated nanocomposite films.

Figure 6 illustrates the mechanism/process according to the preparation method and the morphology results originated from the XRD, FTIR, and SEM measurements of the ZnO@Mt and ZnO@HNT nanohybrids as well as the CS/PVOH/ZnO@Mt and CS/PVOH/ZnO@HNT nanocomposite films.

### 3.4. Tensile Measurements

**Figure 7:**
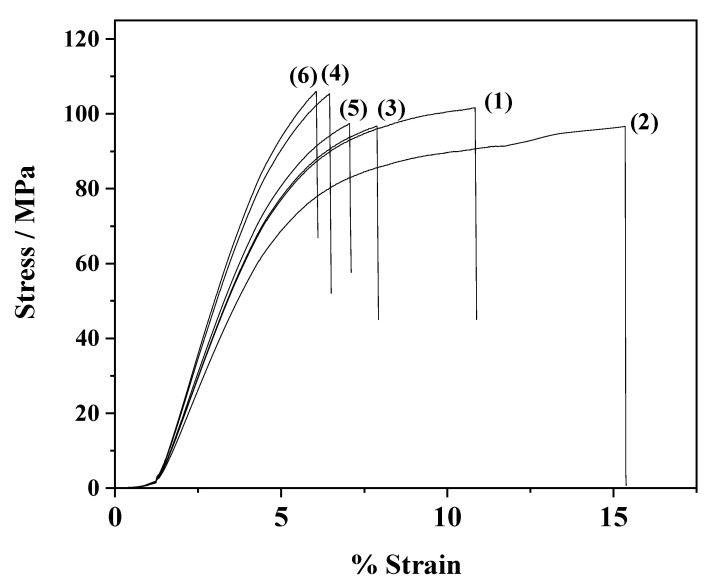
Stress–strain curves of pure CS film (1), CS/PVOH film (2), CS/PVOH/3ZnO@Mt film (3), CS/PVOH/6ZnO@HNT film (4), CS/PVOH/3ZnO@Mt film (5), and CS/PVOH/6ZnO@HNT film (6).

Typical stress–strain curves for pure CS and CS/PVOH films as well as for CS/PVOH/xZnO@Mt and CS/PVOH/xZnO@HNT nanocomposite films are presented in Figure 3. The average values of the E modulus, tensile strength, and strain at break of all tested samples are provided in Table 2. It is obvious in Figure 7 that most of the tested films present a stress–strain behavior typical of a semi-crystalline polymer while the addition of PVOH led to a distinct plastic flow region with higher strain at break values and a pronounced decrease in stiffness and strength. This result is in accordance with others reported in our previous work [26].

The addition of ZnO@Mt or ZnO@HNT nanohybrids provides a pronounced enhancement of the stiffness and strength followed by a significant decrease in the strain at break of the nanocomposite films. The higher the wt% addition of ZnO@Mt or ZnO@HNT nanohybrids, the higher the enhancement of stiffness and strength and the lower the strain at break values. The increase in stiffness and strength values of CS films after adding 2D (Mt) and 1D (HNT) nanofillers was expected and agrees with previous reports where CS/Mt [18,19,26,61,62] and CS/HNT [11,21] nanocomposites were prepared. Ding et al. [38] also recorded a similar increase in tensile strength (approx. 35%) when palygorskite@ZnO nanorods were added to CS films. According to this work, this event could be attributed to the fine dispersion of the nanohybrid and to the hydrogen bond network which developed between the polymer chains and the nanofiller. The increase in stiffness and strength is higher in the case of ZnO@HNT nanocomposites compared to the respective ZnO@Mt nanocomposites. The higher stiffness and strength of Mt-based nanocomposites compared to the corresponding HNT-based nanocomposites is reported for the first time in this work.

### 3.5. Water Sorption

Water sorption values of all tested films are listed in Table 3. It is obvious from the measured data that PVOH, ZnO@Mt, and ZnO@HNT addition results in slightly increased water adsorption values. This increase could be attributed to the excess amount of hydroxyl groups (OH) in the CS/PVOH blends [63,64] and to the uncovered O or OH adsorption sites in NaMt and HNT clay, which readily interact with water [62].

It is worth noting that the total amount of adsorbed water in all films is significantly lower than the amount reported by other researchers [26,63]. This reduction could be associated with the heat pressing process followed for the film formation and could be attributed either to the denser packing of the CS chains or the elimination of pores [16,26].

### 3.6. Water and Oxygen Barrier

WVTR and OTR measured values, as well as calculated D_WV_ and Pe_O2_ values of all the tested films, are listed in Table 3. It can be observed that PVOH addition leads to a decrease in both WVTR and OTR values of the CS/PVOH blend, which could be attributed to the formation of CS-PVOH intermolecular hydrogen bonds. Such bonds limit intermolecular chain mobility [26]. The addition of ZnO@Mt and ZnO@HNT leads to an additional enhancement of both the water and oxygen barrier of the CS/PVOH-based nanocomposites. CS/PVOH/xZnO@HNT nanocomposites exhibited higher water and oxygen barrier properties than CS/PVOH/xZnO@Mt. The increase in water/oxygen barrier reported in this study is in agreement with previous reports where an increase in water/oxygen barrier was observed with the addition of ZnO [29,33,34] or Mt [20,26,27] nanoparticles into the chitosan matrix. In the study of Ding et al. [38], no water and oxygen barrier properties are reported. Thus, the increase in water/oxygen barrier properties when such ZnO@HNT and ZnO@Mt hybrid nanostructures are added to a CS matrix is reported for the first time in this work. Nevertheless, values of WVTR and OTR accompanied by values of film thickness and %RH gradient for PP, HDPE, LDPE, PLA, and CS polymer films were reported in the literature [65,66,67,68,69]. Corresponding values of D_WV_ and Pe_O2_ which were calculated according to the previously mentioned methodology are presented in Table 4.

It is obvious from Table 3 and Table 4 that the materials developed in this work exhibit a higher oxygen barrier (lower oxygen permeability coefficient) compared to the previously reported polymer films, while in all cases the water vapor barrier is lower (higher water vapor diffusion coefficients)

### 3.7. UV–Vis Film Transmittance

**Figure 8:**
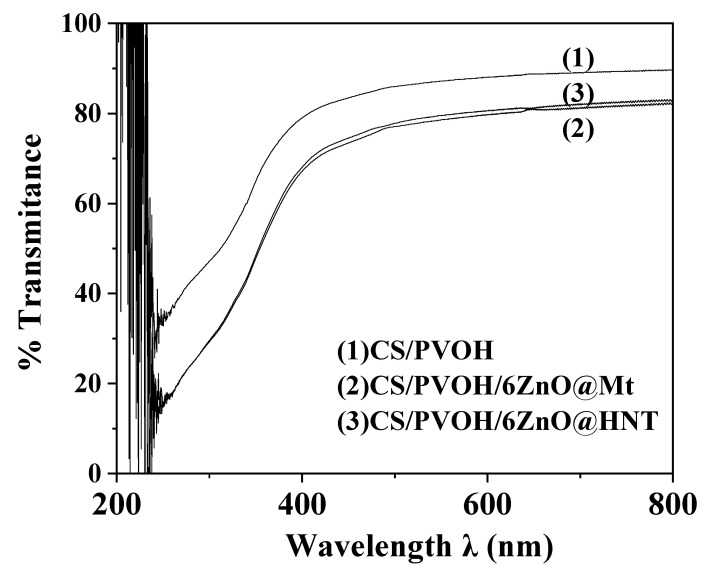
UV–vis % transmittance of (1) CS/PVOH, (2) CS/PVOH/6ZnO@Mt, and (3) CS/PVOH/6ZnO@HNT in the range of 200 to 800 nm.

Representative UV–vis spectra plots in the range of 200 to 800 nm are shown in Figure 8 for CS/PVOH, CS/PVOH/6ZnO@Mt, and CS/PVOH/6ZnO@HNT films. All films exhibited low transmittance in the pure UV range (i.e., 200–300 nm) and high transmittance in the pure visible range (i.e., 600–800 nm). The pure CS/PVOH film exhibited higher transmittance compared to the transmittance of the other two samples in the entire range. This result occurs because of the presence of hybrid ZnO@Mt and ZnO@HNT nanostructures in the last two films and is in accordance with previous reports [38,39].

Moreover, the lower transmittance values of the CS/PVOH/6ZnO@Mt and the CS/PVOH/6ZnO@HNT nanocomposite films are due to the presence of ZnO nanorods and/or Zn^+2^ ions in the film. According to previous reports, this decrease is beneficial for active food packaging films [39].

### 3.8. Antimicrobial Activity

The antibacterial effect of the nanoreinforcement chitosan CS/PVOH-based packaging films which were studied in this work is shown in Table 5.

The films were tested for their antimicrobial efficacy against four food pathogenic bacteria: *Escherichia coli, Staphylococcus aureus, Salmonella enterica,* and *Listeria monocytogenes*. The inhibitory activity was evaluated by measuring the diameter of the clear inhibition zone. When no surrounding clear zone was observed, it was assumed that there is no inhibitory zone, so the diameter was defined as zero. Furthermore, the bacteria growth inhibition under the film discs in direct contact with the agar surface was also evaluated.

It is well known that chitosan has some antimicrobial activities which are attributed to the interaction of the positively charged ammonium (NH_4_^+^) of the amino glucose units with the negatively charged compounds of the bacteria cell wall. Because of this interaction, the bacterial outer membrane breaks down, causing the leakage of essential intracellular constituents and negatively impacting the function of bacterial cells, leading to the death of the cells [70]. However, pure CS possesses poor mechanical properties, and for this problem to be overcome, it is blended with other polymers such as PVOH. Moreover, CS also seems to lose its antibacterial activity in non-acidic conditions, while PVOH exhibits resistance to both acidic and alkaline conditions [71]. Thus, this polymer blend leads to the improvement of the mechanical and, in most cases, antimicrobial properties of the final film. As for neat PVOH, no antimicrobial activity has been previously reported; however, Bonilla et al. (2014) [72] found a reduction in microbial counts of minced pork samples coated only with PVOH films when compared to the control uncoated samples. This reduction could be explained by the strong oxygen barrier of PVOH, which could lead to microbial growth inhibition [73]. In our study, it was noted that CS/PVOH films exhibited better antimicrobial activity in comparison to CS films. The medium used for antimicrobial assays was Mueller–Hinton agar with a pH adjusted close to 7.3. Consequently, CS films did not show the expected antimicrobial activity since chitosan in these conditions has poor solubility and can act as a nutrient for bacteria growth [74]. Moreover, the cross-linking with the PVOH in CS/PVOH films may render the chitosan unapproachable from the bacteria, allowing it to exhibit its antimicrobial activity. Moreover, a polymer, in order to establish antimicrobial activity, should interact with the pathogens dispersed in a water base substrate. Hence, its swelling behavior is a crucial property for its application [75]. Chitosan/PVOH films seem to have the maximum swelling behavior at pH 7 [33] where their antimicrobial activity was observed during our study.

The behaviors of the studied CS/PVOH/3ZnO@Mt, CS/PVOH/6ZnO@Mt, CS/PVOH/3ZnO@HNT, and CS/PVOH/6ZnO@HNT CS films were compared to the relevant CS and CS/PVOH films. Concerning the surrounding clear zone, the chitosan films which were used as control did not show any migrated inhibitory activity against *E. coli*, *S. aureus,* and, *S. enterica*. However, they did show an antibacterial effect underneath the films where no bacterial growth was observed. Moreover, the CS films did not exhibit any antibacterial activity against *L. monocytogenes* in the contact area or by the formation of clear surrounding zones. Furthermore, the CS/PVOH film exhibited antibacterial activity, forming an inhibitory zone of 3.90 mm for *E. coli*, 4.10 mm for *S. aureus,* and 3.00 mm for *S. enterica*, while there was no antimicrobial effect against *L. monocytogenes*. In all cases, the use of the ZnO@Mt and ZnO@HNT nanostructures increased the antibacterial efficacy of the tested films. In most cases, this increment seems to be proportional to the increased addition of the nanostructures. Specifically, the CS/PVOH/6ZnO@Mt film exhibited higher antibacterial activity against *E. coli* and *S. enterica* compared to CS/PVOH/3ZnO@Mt film. On the contrary, CS/PVOH/3ZnO@Mt films showed significantly higher antimicrobial activity against *S. aureus* and *L. monocytogenes* compared to the activity of the CS/PVOH/6ZnO@Mt. This contradiction could be explained based on previous literature reports [76]. According to these reports, zinc ions are essential for normal bacterial cell function, and because of this, the intracellular metabolic processes of bacteria may be stimulated when low concentrations of ZnO are present, while high concentrations of zinc are toxic for bacterial cells. However, zinc plays a complex and contradictory role in bacterial behavior by affecting bacterial pathogenesis, biofilm formation, intracellular growth, etc., differently. Thornton et al. (2017) [77] found that the increased zinc availability could enhance the initial aggregation of Streptococcus pneumoniae and its biofilm formation as well. Specifically, it was shown that in the presence of high zinc concentrations, the bacteria formed more substantial cell clusters and that this behavior is zinc-dependent. In the case of *Staphylococcus aureus*, evidence has shown the importance of zinc presence for the interaction of a surface protein (SasG) responsible for bacteria adhesive function during biofilm formation [78]. Thus, it is possible that the higher antimicrobial activity noted in the case of *S. aureus* and *L. monocytogenes* when lower ΖnO amounts were used is due to the stimulation of biofilm formation. In addition, in the literature, it is found that montmorillonite could enhance the growth of some bacteria. Specifically, Cai et al. (2018) [79] showed that montmorillonite stimulated the growth of *E. coli.* Similar results are also noted in other studies. Respiration of bacteria was found to be promoted by montmorillonite, probably because of the clay’s ability to maintain the pH at levels sufficient for sustained growth [80], while hydrocarbon-degrading bacteria were also stimulated significantly by the presence of this clay [81]. Furthermore, montmorillonite appears to affect the activity of antimicrobial agents such as antibiotics. The antimicrobial activity of tetracycline was found to be suppressed when loaded on montmorillonite, as the growth of non-resistant bacteria was not reduced even when extremely high tetracycline doses were used [82,83]. By reviewing all the above information, it is apparent that the effectiveness of the films studied in the present work depends on numerous mechanisms. Consequently, many parameters appear to be involved, including the kind and the amount of the antimicrobial agent, the interaction between the agent and the clay, the interaction between the microorganism and the clay, the growth media conditions (e.g., pH) and type, the nanoparticle type/size, the bacterial strain, and the bacterial cell concentration.

Considering the HNT-based films, the CS/PVOH/6ZnO@HNT film demonstrated higher antibacterial activity against all tested bacteria compared to the CS/PVOH/3ZnO@HNT film. The inhibitory zones were noticeably higher for CS/PVOH/xZnO@HNT films. The CS/PVOH/3ZnO@HNT film inhibited all the tested bacteria by forming clear zones of 6.00 mm for *E. coli*, 4.25 mm for *S. aureus*, 4.00 mm for *S. enterica,* and 3.50 mm for *L. monocytogenes.* The CS/PVOH/6ZnO@HNT film exhibited an inhibitory zone of 8.50 mm for *E. coli*, 5.50 mm for *S. aureus*, 4.50 mm for *S. enterica,* and 4.51 mm for *L. monocytogenes*. By reviewing the results and comparing the antibacterial activity of the nanoreinforcement films, it is obvious that there are significant differences. Both of the CS/PVOH/xZnO@HNT films exhibited statistically significant antibacterial activity against all tested bacteria compared to the CS films, while CS/PVOH/6ZnO@HNT films showed statistically higher antibacterial activity against all tested bacteria compared to the CS/PVOH films. Moreover, in all cases, the nanoreinforcement films exhibited better antimicrobial properties in comparison to CS/PVOH, except the CS/PVOH/6ZnO@Mt which showed lower antimicrobial activity against *S. aureus* and *L. monocytogenes*. Natural clay minerals such as HNT and Mt are widely used for many applications by loading different nanomaterials. Specifically, they were used for their ability to improve the dispersal of ZnO in nanostructures, their unique ion exchange capacity, their hydrophilicity, and their exceptional mechanical properties [84]. In this work, it is obvious that the use of these clays in combination with ΖnO as reinforcements of CS films showed great results as far as the antimicrobial activity was concerned. As mentioned before, the antimicrobial activity of CS has been well recognized for many years, and in combination with the properties of PVOH and/or other reinforcements, the new hybrid materials will offer new characteristics [85,86].

In the present study, films incorporated with ZnO and two different types of clays (Mt and HNT) demonstrated better results compared with CS films. ZnO nanoparticles seem to exhibit a wide range of antibacterial activities against both Gram-positive and Gram-negative bacteria, including foodborne pathogens such as *E. coli*, *Salmonella enterica spp.*, *L.* *monocytogenes*, and *S. aureus* [87,88] The use of clays in matrices such as CS/PVOH/xZnO@clays seems to affect the antimicrobial properties of the final nanomaterial positively by bringing ZnO nanoparticles closer to the membrane of bacteria and leading to the blockage of the normal bacteria function [89]. Moreover, it was mentioned that the presence of clays increases the local zinc concentration, causing inhibition of the bacteria growth [90]. Specifically, HNT seems to facilitate the dispersion and stability of ZnO nanoparticles and control their distribution. As mentioned in the XRD section, the ZnO nanorods exhibit lower crystal size in the case of ZnO@HNT nanostructure than in the case of ZnO@Mt nanostructure. In this way, ZnO nanoparticles have better and closer contact with the bacteria cells, leading to the easiest interruption of their membrane functions. Moreover, it seems that the bacteria growth inhibition is correlated to the increased nanocomposite concentration. A possible explanation for this antibacterial activity is that when the nanoparticles were attached better to the bacteria membrane, they produced an important level of reactive oxygen species created by hydroxyl radicals under visible light. Thus, when ZnO nanoparticles exhibit higher dispersion, it is possible to offer a bigger external surface area and sequentially more active sites to produce more reactive oxygen species. Then, singlet oxygen could oxidize the cell content, finally causing bacterial disorganization [84].

Montmorillonite can also adsorb and carry heavy metal ions. Consequently, its antibacterial activities could also be attributed to the interactions of these metal ions with the bacteria. Moreover, the presence of montmorillonite clays in matrices such as CS/PVOH appears to affect their antimicrobial activity positively, and by increasing the clay content, the bacteria growth inhibition is increased accordingly [86,91]. These theories are in accordance with the results of the present study, which show that the incorporation of halloysite and montmorillonite in CS/PVOH/xZnO matrices caused higher antimicrobial activity for most of the cases studied.

However, it is worth mentioning that the final effect of the studied films against the tested bacteria is obviously due to the synergistic action of CS, PVOH, and nanoclay. In addition, the antimicrobial activity of any nanostructure is also associated with the bacterial strain, nanoparticle type/size, growth media type, and bacterial cell concentration [92].

### 3.9. Statistical Analysis of the Experimental Data

All numbers in Table 2, Table 3 and Table 4 represent the mean values of properties. At least three to five different samples were tested to estimate the final value of each quantity. Standard deviation values are also reported in the tables. The inequality of mean values between all kinds of samples was statistically checked via the statistical hypothesis H0 (mean values could be assumed as equal). This test was performed for supporting the hypothesis that every property of different kinds of samples exhibited a statistically different mean value. SPSS ver. 20 software was used to statistically treat the experimental values of E, σ_uts_, ε_b_%, WVTR,% water sorption, OTR, *E. coli*, *S. aureus*, *S. enterica*, and *L. monocytogenes.* The common value C.I. = 95% was adopted for the confidential intervals, and thus a significance level of a = 0.05 was assumed. The overall procedure and the relevant equations are detailly reported in [28]. The nonparametric Mann–Whitney U test for independent samples was used instead of the ANOVA test because tests indicated non-normality in some datasets. Moreover, “inequality assurance” and “equality assurance” calculations [28] were carried out. In all cases, the hypothesis H0 was found to be false, and the “inequality assurance” of the unequal mean values was IA > 88%.

## 4. Conclusions

It was confirmed by the antimicrobial measurements of this work that the ZnO@clay nanostructure significantly enhances the antimicrobial activity of the CS/PVOH films. This happens because of the presence of the ZnO material which partially grew as well-oriented nanorods on the external surface of the clay and partially dissolved as Zn^2+^ ions in the CS matrix. This is evidenced by both the XRD and the SEM-EDX measurements and analysis. Of the two nanohybrid materials, i.e., the ZnO@Mt and the ZnO@HNT, the latter exhibited more advanced antimicrobial properties. This was expected because of the smaller crystal size which was calculated by the XRD measurements and the over double Zn content which was estimated by SEM-EDX measurements. It was also shown by the XRD spectra that CS/PVOH composite material intercalates clay, providing final films with a perfectly homogeneous structure, as is indicated by the FT-IR measurements and the SEM photos. These films exhibited lower water vapor diffusion coefficient and oxygen permeability values, indicating increased barrier properties compared with the relevant CS and CS/PVOH films. Nevertheless, they exhibit higher oxygen barrier and lower water vapor barrier properties compared to other polymeric films. This is normal due to the hydrophilicity of chitosan. They also exhibited improved mechanical properties, as shown by the tensile measurements. Such improved mechanical properties could be attributed to the fine dispersion of the nanohybrid, which is obvious from the SEM images. Moreover, they could be attributed to the hydrogen bond network between the nanofiller and the polymer chains. In conclusion, the CS/PVOH/6ZnO@HNT produced by following the procedure proposed by this study for the synthesis of CS/PVOH/xZnO@clay films is a promising material for active food packaging applications.

## Figures and Tables

**Table 1:** Code name and amounts of CS, PVOH, ZnO@Mt, and ZnO@HNT used for the preparation of all CS/PVOH/xZnO@Mt ^1^ and CS/PVOH/xZnO@HNT ^1^ active films.

Code Name	CS gr-wt%	PVOH gr-wt%	ZnO@Mt gr-wt%	ZnO@HNT gr-wt%
CS	2.0–100	-	-	-
CS/PVOH	2.0–100	0.25–20	-	-
CS/PVOH/3ZnO@Mt	2.0–100	0.5–20	0.08–3	-
CS/PVOH/6ZnO@Mt	2.0–100	0.59	0.17–6	-
CS/PVOH/3ZnO@HNT	2.0–100	0.5–20	-	0.08–3
CS/PVOH/6ZnO@HNT	2.0–100	0.59–20	-	0.17–6

^1^ CS/PVOH/xZnO@clay film contains x% ZnO@clay nanostructure in CS/PVOH matrix.

**Table 2:** Calculated values of Young’s (E) modulus, ultimate tensile strength (_uts), and % strain at break (“b).

Code Name	E Modulus (MPa)	σ_uts_ (MPa)	ε_b_%
CS	3274 ± 62	92.2 ± 3.5	10.8 ± 1.4
CS/PVOH	2920 ± 105	86.4 ± 3.4	14.5 ± 0.8
CS/PVOH/3ZnO@Mt	3300 ± 83	93.8 ± 4.2	7.9 ± 1.7
CS/PVOH/6ZnO@Mt	3840 ± 51	106.4 ± 2.8	6.4 ± 10.2
CS/PVOH/3ZnO@HNT	3430 ± 89	95.8 ± 4.2	7.1 ± 1.8
CS/PVOH/6ZnO@HNT	3980 ± 51	109.1 ± 2.8	6.1 ± 1.2

**Table 3:** Experimentally measured water vapor transmission rate (WVTR) and oxygen transmission rate (OTR) values and calculated water vapor diffusion coefficient (D_WV_) and oxygen permeability coefficient (Pe_O2_) values.

Sample Code	Mean Value ± Std. Err. Film Thick. (mm)	Mean Value ± Std. Err. % Water Sorption	Mean Value ± Std. Err. WVTR (g/cm^2^·Day)	D_WV_ (10^−4^ cm^2^/s)	Mean Value ± Std. Err. OTR (mL/m^2^·Day)	Pe_O2_ (10^−10^ cm^2^/s)
CS	0.15 ± 0.002	175 ± 4	1.1764 ± 0.0102	89.313	40.1 ± 1.6	6.9618
CS/PVOH	0.17 ± 0.003	184 ± 5	0.1613 ± 0.0097	13.879	38.2 ± 1.9	7.5162
CS/PVOH/3ZnO@Mt	0.16 ± 0.002	191 ± 6	0.1521 ± 0.0091	12.317	26.9 ± 1.3	4.9815
CS/PVOH/6ZnO@Mt	0.13 ± 0.003	194 ± 5	0.1384 ± 0.0083	9.106	21.5 ± 1.1	3.2350
CS/PVOH/3ZnO@HNT	0.14 ± 0.001	190 ± 4	0.1452 ± 0.0087	10.289	19.5 ± 0.9	3.1597
CS/PVOH/6ZnO@HNT	0.15 ± 0.002	192 ± 7	0.1120 ± 0.0067	8.503	14.4 ± 0.4	2.5000

**Table 4:** Calculated D_WV_ and Pe_O2_ values based on WVTR and OTR measurements of previously reported polymer films.

Sample Code	Mean Value Film Thick. (mm)	Mean Value WVTR (g·m^−2^·day^−1^)	D_WV_ (cm^2^·s^−1^)	Mean Value OTR (cm^3^·m^−2^·day^−1^)	Pe_O2_ (cm^2^·s^−1^)
PP [65]	0.0254	3.9	2.76 × 10^−7^	-	-
PP [66]	0.04	1.4	1.12 × 10^−5^	2702	1.25 × 10^−8^
HDPE [65]	0.0254	4.7–7.8	(3.32–5.52) 10^−7^	-	-
LDPE [65]	0.0254	16–23	(1.13–1.63) 10^−6^	-	-
LDPE [66]	0.05	5.6	5.61 × 10^−5^	6314	3.65 × 10^−8^
LDPE [67]	0.03175	2.66–16.29	(5.65–11.73) × 10^−7^	-	-
PLA [68]	0.15–0.35	195.1–103	(1.94–2.39) × 10^−4^	1400–377	(4.21–5.76) × 10^−8^
CS [69]	0.02	1200	6.77 × 10^−5^	-	-

**Table 5:** Antimicrobial activity of active films against food pathogenic bacteria *E. coli*, *S. aureus*, *S. enterica*, and *L. monocytogenes*.

Film Material	*E. coli*		*S. aureus*		*S. enterica*		*L. monocytogenes*	
	Inhibition ^1^ (Mean Diameter of Clear Zone)	Contact ^2^ (Under the Film Disc)	Inhibition ^1^ (Mean Diameter of Clear Zone)	Contact ^2^ (Under the Film Disc)	Inhibition ^1^ (Mean Diameter of Clear Zone)	Contact ^2^ (Under the Film Disc)	Inhibition ^1^ (Mean Diameter of Clear Zone)	Contact ^2^ (Under the Film Disc)
CS	0.00 ^a^	-	0.00 ^a^	-	0.00 ^a^	-	0.00 ^a^	+
CS/PVOH	3.90 ± 0.14 ^b^	-	4.10 ± 0.14 ^b,c^	-	3.00 ± 0.00 ^b^	-	0.00 ^a^	+
CS/PVOH/3ZnO@Mt	4.04 ± 1.41 ^b^	-	5.00± 0.00 ^d,e^	-	4.00 ± 0.00 ^b,c^	-	2.00 ± 0.00 ^b^	-
CS/PVOH/6ZnO@Mt	5.00 ± 0.00 ^b,c^	-	3.50 ± 0.71 ^b^	-	4.50 ± 0.71 ^c,d^	-	0.00 ^a^	-
CS/PVOH/3ZnO@HNT	6.00 ± 0.72 ^c^	-	4.25 ± 0.35 ^b,c^	-	4.00 ± 0.01 ^b,c^	-	3.50 ± 0.71 ^c^	-
CS/PVOH/6ZnO@HNT	8.50 ± 0.71 ^d^	-	5.50 ± 0.71 ^e^	-	4.50 ± 0.71 ^c,d^	-	4.51 ± 0.71 ^c^	-

^1^ Inhibitory zone surrounding film discs measured in mm after the subtraction of the disc diameter (6 mm). ^2^ Contact area of film discs with the agar surface. (+) indicates bacterial growth in the area, (-) indicates no bacterial growth in the area. Results expressed as mean value ± standard error (*n* = 3). Means in the same column showing different superscript letters are significantly different (*p* < 0.5).

## Data Availability

The datasets generated for this study are available on request to the corresponding author.

## References

[1] Shamshina J.L., Berton P., Rogers R.D. (2019). Advances in Functional Chitin Materials: A Review. ACS Sustain. Chem. Eng..

[2] Cazón P., Vázquez M., Crini G., Lichtfouse E. (2019). Applications of Chitosan as Food Packaging Materials. Sustainable Agriculture Reviews 36: Chitin and Chitosan: Applications in Food, Agriculture, Pharmacy, Medicine and Wastewater Treatment.

[3] Aider M. (2010). Chitosan Application for Active Bio-Based Films Production and Potential in the Food Industry: Review. LWT Food Sci. Technol..

[4] Elsabee M.Z., Abdou E.S. (2013). Chitosan Based Edible Films and Coatings: A Review. Mater. Sci. Eng. C.

[5] Ravi Kumar M.N.V. (2000). A Review of Chitin and Chitosan Applications. React. Funct. Polym..

[6] Younes I., Rinaudo M. (2015). Chitin and Chitosan Preparation from Marine Sources. Structure, Properties and Applications. Mar. Drugs.

[7] Haghighi H., Licciardello F., Fava P., Siesler H.W., Pulvirenti A. (2020). Recent Advances on Chitosan-Based Films for Sustainable Food Packaging Applications. Food Packag. Shelf Life.

[8] Tang C., Chen N., Zhang Q., Wang K., Fu Q., Zhang X. (2009). Preparation and Properties of Chitosan Nanocomposites with Nanofillers of Different Dimensions. Polym. Degrad. Stab..

[9] Bumbudsanpharoke N., Ko S. (2019). Nanoclays in Food and Beverage Packaging. J. Nanomater..

[10] Giannakas E.A., Leontion A. (2018). Montmorillonite Composite Materials and Food Packaging. Composites Materials for Food Packaging.

[11] Barman M., Mahmood S., Augustine R., Hasan A., Thomas S., Ghosal K. (2020). Natural Halloysite Nanotubes /Chitosan Based Bio-Nanocomposite for Delivering Norfloxacin, an Anti-Microbial Agent in Sustained Release Manner. Int. J. Biol. Macromol..

[12] Applications of Halloysite Nanotubes in Food Packaging for Improving Film Performance and Food Preservation—ScienceDirect. https://www.sciencedirect.com/science/article/pii/S0956713521000141?via%3Dihub.

[13] Rhim J.W., Park H.M., Ha C.S. (2013). Bio-Nanocomposites for Food Packaging Applications. Prog. Polym. Sci..

[14] Shankar S., Rhim J.-W. (2018). Bionanocomposite Films for Food Packaging Applications. Reference Module in Food Science.

[15] Haerudin H., Pramono A.W., Kusuma D.S., Jenie A., Voelcker N.H., Gibson C. (2010). Preparation and Characterization of Chitosan/Montmorillonite (MMT) Nanocomposite Systems. Int. J. Technol..

[16] Giannakas A., Grigoriadi K., Leontiou A., Barkoula N.-M., Ladavos A. (2014). Preparation, Characterization, Mechanical and Barrier Properties Investigation of Chitosan–Clay Nanocomposites. Carbohydr. Polym..

[17] Grigoriadi K., Giannakas A., Ladavos A.K., Barkoula N.-M. (2015). Interplay between Processing and Performance in Chitosan-Based Clay Nanocomposite Films. Polym. Bull..

[18] Dias M.V., Azevedo V.M., Borges S.V., Soares N.D.F.F., Fernandes R.V.D.B., Marques J.J., Medeiros A.A. (2014). Development of Chitosan/Montmorillonite Nanocomposites with Encapsulated α-Tocopherol. Food Chem..

[19] Gomes V., Souza L., Pires J.R.A., Freitas P., Lopes A.A.S., Fernandes F.M.B., Paula M., Coelhoso I.M., Luisa A. (2018). Bionanocomposites of Chitosan / Montmorillonite Incorporated with Rosmarinus o Ffi Cinalis Essential Oil: Development and Physical Characterization. Food Packag. Shelf Life.

[20] Casariego A., Souza B.W.S., Cerqueira M.A., Teixeira J.A., Cruz L., Díaz R., Vicente A.A. (2009). Chitosan/Clay Films’ Properties as Affected by Biopolymer and Clay Micro/Nanoparticles’ Concentrations. Food Hydrocoll..

[21] Huang D., Zhang Z., Zheng Y., Quan Q., Wang W., Wang A. (2020). Synergistic Effect of Chitosan and Halloysite Nanotubes on Improving Agar Film Properties. Food Hydrocoll..

[22] Wang Y., Yi S., Lu R., Sameen D.E., Ahmed S., Dai J., Qin W., Li S., Liu Y. (2021). Preparation, Characterization, and 3D Printing Verification of Chitosan/Halloysite Nanotubes/Tea Polyphenol Nanocomposite Films. Int. J. Biol. Macromol..

[23] Jauković V., Krajišnik D., Daković A., Damjanović A., Krstić J., Stojanović J., Čalija B. (2021). Influence of Selective Acid-Etching on Functionality of Halloysite-Chitosan Nanocontainers for Sustained Drug Release. Mater. Sci. Eng. C.

[24] Pasbakhsh P., Silva R.D., Vahedi V., Churchman G.J. (2016). Halloysite Nanotubes: Prospects and Challenges of Their Use as Additives and Carriers—A Focused Review. Clay Miner..

[25] Saadat S., Pandey G., Tharmavaram M., Braganza V., Rawtani D. (2020). Nano-Interfacial Decoration of Halloysite Nanotubes for the Development of Antimicrobial Nanocomposites. Adv. Colloid Interface Sci..

[26] Giannakas A., Vlacha M., Salmas C., Leontiou A., Katapodis P., Stamatis H., Barkoula N.-M., Ladavos A. (2016). Preparation, Characterization, Mechanical, Barrier and Antimicrobial Properties of Chitosan/PVOH/Clay Nanocomposites. Carbohydr. Polym..

[27] Boesel L.F. (2015). Effect of Plasticizers on the Barrier and Mechanical Properties of Biomimetic Composites of Chitosan and Clay. Carbohydr. Polym..

[28] Salmas C.E., Giannakas A.E., Baikousi M., Kollia E., Tsigkou V., Proestos C. (2021). Effect of Copper and Titanium-Exchanged Montmorillonite Nanostructures on the Packaging Performance of Chitosan/Poly-Vinyl-Alcohol-Based Active Packaging Nanocomposite Films. Foods.

[29] Boura-Theodoridou O., Giannakas A., Katapodis P., Stamatis H., Ladavos A., Barkoula N.-M. (2020). Performance of ZnO/Chitosan Nanocomposite Films for Antimicrobial Packaging Applications as a Function of NaOH Treatment and Glycerol/PVOH Blending. Food Packag. Shelf Life.

[30] Kumar R., Umar A., Kumar G., Nalwa H.S. (2017). Antimicrobial Properties of ZnO Nanomaterials: A Review. Ceram. Int..

[31] Roohani N., Hurrell R., Kelishadi R., Schulin R. (2013). Zinc and Its Importance for Human Health: An Integrative Review. J. Res. Med. Sci..

[32] Boyer R.F. (1983). A Review of: “Zinc and Its Role in Biology and Nutrition, Volume 15 of Metal Ions in Biological Systems, H. Sigel, Editor, Xxii + 493 Pages, Marcel Dekker, Inc., New York, 1983”. Synth. React. Inorg. Metal. Org. Chem..

[33] Abdeen Z.I., El Farargy A.F., Negm N.A. (2018). Nanocomposite Framework of Chitosan/Polyvinyl Alcohol/ZnO: Preparation, Characterization, Swelling and Antimicrobial Evaluation. J. Mol. Liq..

[34] Al-Naamani L., Dobretsov S., Dutta J. (2016). Chitosan-Zinc Oxide Nanoparticle Composite Coating for Active Food Packaging Applications. Innov. Food Sci. Emerg. Technol..

[35] Bano I., Arshad M., Yasin T., Ghauri M.A. (2019). Preparation, Characterization and Evaluation of Glycerol Plasticized Chitosan/PVA Blends for Burn Wounds. Int. J. Biol. Macromol..

[36] Murali S., Kumar S., Koh J., Seena S., Singh P., Ramalho A., Sobral A.J.F.N. (2019). Bio-Based Chitosan/Gelatin/Ag@ZnO Bionanocomposites: Synthesis and Mechanical and Antibacterial Properties. Cellulose.

[37] Akkari M., Aranda P., Ben Rhaiem H., Ben Haj Amara A., Ruiz-Hitzky E. (2016). ZnO/Clay Nanoarchitectures: Synthesis, Characterization and Evaluation as Photocatalysts. Appl. Clay Sci..

[38] Ding J., Hui A., Wang W., Yang F., Kang Y., Wang A. (2021). Multifunctional Palygorskite@ZnO Nanorods Enhance Simultaneously Mechanical Strength and Antibacterial Properties of Chitosan-Based Film. Int. J. Biol. Macromol..

[39] Salmas C., Giannakas A., Katapodis P., Leontiou A., Moschovas D., Karydis-Messinis A. (2020). Development of ZnO/Na-Montmorillonite Hybrid Nanostructures Used for PVOH/ZnO/Na-Montmorillonite Active Packaging Films Preparation via a Melt-Extrusion Process. Nanomaterials.

[40] Jia W., Dang S., Liu H., Zhang Z., Yu C., Liu X., Xu B. (2012). Evidence of the Formation Mechanism of ZnO in Aqueous Solution. Mater. Lett..

[41] Giannakas A., Patsaoura A., Barkoula N.-M., Ladavos A. (2017). A Novel Solution Blending Method for Using Olive Oil and Corn Oil as Plasticizers in Chitosan Based Organoclay Nanocomposites. Carbohydr. Polym..

[42] Vlacha M., Giannakas A., Katapodis P., Stamatis H., Ladavos A., Barkoula N.-M. (2016). On the Efficiency of Oleic Acid as Plasticizer of Chitosan/Clay Nanocomposites and Its Role on Thermo-Mechanical, Barrier and Antimicrobial Properties—Comparison with Glycerol. Food Hydrocoll..

[43] Giannakas A., Giannakas A., Ladavos A. (2012). Preparation and Characterization of Polystyrene/Organolaponite Nanocomposites. Polym. Plast. Technol. Eng..

[44] Giannakas A., Salmas C., Leontiou A., Tsimogiannis D., Oreopoulou A., Braouhli J. (2019). Novel LDPE/Chitosan Rosemary and Melissa Extract Nanostructured Active Packaging Films. Nanomaterials.

[45] Giannakas Na-Montmorillonite V.S. (2020). Organically Modified Montmorillonite as Essential Oil Nanocarriers for Melt-Extruded Low-Density Poly-Ethylene Nanocomposite Active Packaging Films with a Controllable and Long-Life Antioxidant Activity. Nanomaterials.

[46] Xu F., Yuan Z.-Y., Du G.-H., Ren T.-Z., Bouvy C., Halasa M., Su B.-L. (2006). Simple Approach to Highly Oriented ZnO Nanowire Arrays: Large-Scale Growth, Photoluminescence and Photocatalytic Properties. Nanotechnology.

[47] Hsu H.-C., Cheng C.-S., Chang C.-C., Yang S., Chang C.-S., Hsieh W.-F. (2005). Orientation-Enhanced Growth and Optical Properties of ZnO Nanowires Grown on Porous Silicon Substrates. Nanotechnology.

[48] Williamson G.K., Hall W.H. (1953). X-Ray Line Broadening from Filed Aluminium and Wolfram. Acta Metall..

[49] Makarona E., Koutzagioti C., Salmas C., Ntalos G., Skoulikidou M.-C., Tsamis C. (2017). Enhancing Wood Resistance to Humidity with Nanostructured ZnO Coatings. Nano Struct. Nano Objects.

[50] Kim I., Viswanathan K., Kasi G., Thanakkasaranee S., Sadeghi K., Seo J. (2020). ZnO Nanostructures in Active Antibacterial Food Packaging: Preparation Methods, Antimicrobial Mechanisms, Safety Issues, Future Prospects, and Challenges. Food Rev. Int..

[51] Gartner C., López B.L., Sierra L., Graf R., Spiess H.W., Gaborieau M. (2011). Interplay between Structure and Dynamics in Chitosan Films Investigated with Solid-State NMR, Dynamic Mechanical Analysis, and X-ray Diffraction. Biomacromolecules.

[52] Rhim J.-W., Hong S.-I., Park H.-M., Ng P.K.W. (2006). Preparation and Characterization of Chitosan-Based Nanocomposite Films with Antimicrobial Activity. J. Agric. Food Chem..

[53] Naveen Kumar H.M.P., Prabhakar M.N., Venkata Prasad C., Madhusudhan Rao K., Ashok Kumar Reddy T.V., Chowdoji Rao K., Subha M.C.S. (2010). Compatibility Studies of Chitosan/PVA Blend in 2% Aqueous Acetic Acid Solution at 30 °C. Carbohydr. Polym..

[54] Wu L.M., Tong D.S., Zhao L.Z., Yu W.H., Zhou C.H., Wang H. (2014). Fourier Transform Infrared Spectroscopy Analysis for Hydrothermal Transformation of Microcrystalline Cellulose on Montmorillonite. Appl. Clay Sci..

[55] Djomgoue P., Njopwouo D. (2013). FT-IR Spectroscopy Applied for Surface Clays Characterization. J. Surf. Eng. Mater. Adv. Technol..

[56] Giannakas A., Tsagkalias I., Achilias D.S., Ladavos A. (2017). A Novel Method for the Preparation of Inorganic and Organo-Modified Montmorillonite Essential Oil Hybrids. Appl. Clay Sci..

[57] Gaaz T.S., Sulong A.B., Kadhum A.A.H., Al-Amiery A.A., Nassir M.H., Jaaz A.H. (2017). The Impact of Halloysite on the Thermo-Mechanical Properties of Polymer Composites. Molecules.

[58] Anžlovar A., Crnjak Orel Z., Kogej K., Žigon M. Polyol-Mediated Synthesis of Zinc Oxide Nanorods and Nanocomposites with Poly(Methyl Methacrylate). https://www.hindawi.com/journals/jnm/2012/760872/.

[59] Soni B.H., Garg N. (2013). Studies on ZnO Nanorods Synthesized by Hydrothermal Method and Their Characterization. J. Nano-Electron. Phys..

[60] Mallakpour S., Madani M. (2012). Transparent and Thermally Stable Improved Poly (Vinyl Alcohol)/Cloisite Na+/ZnO Hybrid Nanocomposite Films: Fabrication, Morphology and Surface Properties. Prog. Org. Coat..

[61] Giannakas A., Stathopoulou P., Tsiamis G., Salmas C. (2019). The Effect of Different Preparation Methods on the Development of Chitosan/Thyme Oil/Montmorillonite Nanocomposite Active Packaging Films. J. Food Process. Preserv..

[62] Lavorgna M., Piscitelli F., Mangiacapra P., Buonocore G.G. (2010). Study of the Combined Effect of Both Clay and Glycerol Plasticizer on the Properties of Chitosan Films. Carbohydr. Polym..

[63] Hyder M.N., Chen P. (2009). Pervaporation Dehydration of Ethylene Glycol with Chitosan-Poly(Vinyl Alcohol) Blend Membranes: Effect of CS-PVA Blending Ratios. J. Membr. Sci..

[64] Srinivasa P.C., Ramesh M.N., Kumar K.R., Tharanathan R.N. (2003). Properties and Sorption Studies of Chitosan-Polyvinyl Alcohol Blend Films. Carbohydr. Polym..

[65] Gas Barrier Properties Enhancement—ScienceDirect. https://www.sciencedirect.com/science/article/pii/B9780323358842000181?via%3Dihub.

[66] Influence of Packaging Material on Quality Characteristics of Minimally Processed Mridula Pomegranate (Punica Granatum) Arils during Cold Storage|Semantic Scholar. https://www.semanticscholar.org/paper/Influence-of-packaging-material-on-quality-of-arils-Bhatia-Asrey/aad1332359b35ef50b4c67b3e73395343d75373d.

[67] Huff K. (2008). Active and Intelligent Packaging: Innovations for the Future. Ph.D. Thesis.

[68] Water Vapor Transmission Rate of Biomass Based Film Materials. https://www.jstage.jst.go.jp/article/eaef/4/2/4_2_37/_article/-char/en.

[69] Wiles J.L., Vergano P.j., Barron F.h., Bunn J.m., Testin R.f. (2000). Water Vapor Transmission Rates and Sorption Behavior of Chitosan Films. J. Food Sci..

[70] Shahidi F., Arachchi J.K.V., Jeon Y.-J. (1999). Food Applications of Chitin and Chitosans. Trends Food Sci. Technol..

[71] Cazón P., Velazquez G., Ramírez J.A., Vázquez M. (2017). Polysaccharide-Based Films and Coatings for Food Packaging: A Review. Food Hydrocoll..

[72] Bonilla J., Fortunati E., Atarés L., Chiralt A., Kenny J.M. (2014). Physical, Structural and Antimicrobial Properties of Poly Vinyl Alcohol–Chitosan Biodegradable Films. Food Hydrocoll..

[73] Gaume J., Rivaton A., Thérias S., Gardette J.-L. (2012). A Promising Method for Measuring Oxygen Permeability of Polymers Using PEO Photooxidation as a Sensor. J. Membr. Sci..

[74] Qin C., Li H., Xiao Q., Liu Y., Zhu J., Du Y. (2006). Water-Solubility of Chitosan and Its Antimicrobial Activity. Carbohydr. Polym..

[75] Wang T., Zhang F., Zhao R., Wang C., Hu K., Sun Y., Politis C., Shavandi A., Nie L. (2020). Polyvinyl Alcohol/Sodium Alginate Hydrogels Incorporated with Silver Nanoclusters via Green Tea Extract for Antibacterial Applications. Des. Monomers Polym..

[76] Suryawati B. (2018). Zinc Homeostasis Mechanism and Its Role in Bacterial Virulence Capacity. AIP Conf. Proc..

[77] Zinc as an Agent for the Prevention of Biofilm Formation by Pathogenic Bacteria—PubMed. https://pubmed.ncbi.nlm.nih.gov/23509865/.

[78] Zinc-Dependent Mechanical Properties of Staphylococcus Aureus Biofilm-Forming Surface Protein SasG|PNAS. https://www.pnas.org/doi/10.1073/pnas.1519265113.

[79] Cai P., Liu X., Ji D., Yang S., Walker S.L., Wu Y., Gao C., Huang Q. (2018). Impact of Soil Clay Minerals on Growth, Biofilm Formation, and Virulence Gene Expression of *Escherichia Coli* O157:H7. Environ. Pollut..

[80] Influence of Clay Minerals on Microorganisms: I. MONTMORILLONITE and Kaolinite on Bacteria. https://cdnsciencepub.com/doi/10.1139/m66-078.

[81] Montmorillonite Mitigates the Toxic Effect of Heavy Oil on Hydrocarbon-Degrading Bacterial Growth: Implications for Marine Oil Spill Bioremediation|Clay Minerals|Cambridge Core. https://www.cambridge.org/core/journals/clay-minerals/article/abs/montmorillonite-mitigates-the-toxic-effect-of-heavy-oil-on-hydrocarbondegrading-bacterial-growth-implications-for-marine-oil-spill-bioremediation/016843E5C47A5619C14AC0674308B81D.

[82] Lv G., Pearce C.W., Gleason A., Liao L., MacWilliams M.P., Li Z. (2013). Influence of Montmorillonite on Antimicrobial Activity of Tetracycline and Ciprofloxacin. J. Asian Earth Sci..

[83] Parolo M.E., Avena M.J., Pettinari G., Zajonkovsky I., Valles J.M., Baschini M.T. (2010). Antimicrobial Properties of Tetracycline and Minocycline-Montmorillonites. Appl. Clay Sci..

[84] Shu Z., Zhang Y., Yang Q., Yang H. (2017). Halloysite Nanotubes Supported Ag and ZnO Nanoparticles with Synergistically Enhanced Antibacterial Activity. Nanoscale Res. Lett.

[85] Benhabiles M.S., Salah R., Lounici H., Drouiche N., Goosen M.F.A., Mameri N. (2012). Antibacterial Activity of Chitin, Chitosan and Its Oligomers Prepared from Shrimp Shell Waste. Food Hydrocoll..

[86] Butnaru E., Cheaburu C.N., Yilmaz O., Pricope G.M., Vasile C. (2016). Poly(Vinyl Alcohol)/Chitosan/Montmorillonite Nanocomposites for Food Packaging Applications: Influence of Montmorillonite Content. High Perform. Polym..

[87] Jones N., Ray B., Ranjit K.T., Manna A.C. (2008). Antibacterial Activity of ZnO Nanoparticle Suspensions on a Broad Spectrum of Microorganisms. FEMS Microbiol. Lett..

[88] Liu Y., He L., Mustapha A., Li H., Hu Z.Q., Lin M. (2009). Antibacterial Activities of Zinc Oxide Nanoparticles against *Escherichia Coli* O157:H7. J. Appl. Microbiol..

[89] Wei J.-C., Yen Y.-T., Su H.-L., Lin J.-J. (2011). Inhibition of Bacterial Growth by the Exfoliated Clays and Observation of Physical Capturing Mechanism. J. Phys. Chem. C.

[90] Clay: New Opportunities for Tissue Regeneration and Biomaterial Design—PubMed. https://pubmed.ncbi.nlm.nih.gov/23722321/.

[91] Malachová K., Praus P., Rybková Z., Kozák O. (2011). Antibacterial and Antifungal Activities of Silver, Copper and Zinc Montmorillonites. Appl. Clay Sci..

[92] Bagchi B., Kar S., Dey S.K., Bhandary S., Roy D., Mukhopadhyay T.K., Das S., Nandy P. (2013). In Situ Synthesis and Antibacterial Activity of Copper Nanoparticle Loaded Natural Montmorillonite Clay Based on Contact Inhibition and Ion Release. Colloids Surf B Biointerfaces.

